# The Role of the Transjugular Intrahepatic Porto-Systemic Shunt in an Emergency Setting

**DOI:** 10.3390/life13040868

**Published:** 2023-03-24

**Authors:** Alessandro Posa, Lorenzo Tenore, Pierluigi Barbieri, Giulia Mazza, Evis Sala, Roberto Iezzi

**Affiliations:** 1 Department of Diagnostic Imaging, Oncologic Radiotherapy and Hematology, A. Gemelli University Hospital Foundation IRCCS, L.go A. Gemelli 8, 00168 Rome, Italy; 2 Istituto di Radiodiagnostica, Catholic University of the Sacred Heart, L.go F. Vito 1, 00168 Rome, Italy

**Keywords:** TIPSS, emergency, variceal bleeding, encephalopathy, technical difficulties

## Abstract

Transjugular intrahepatic porto-systemic shunt (TIPSS) is an interventional radiology procedure whose aim is to create artificial communication between the portal and the hepatic blood flow in order to reduce the pressure gradient in portal hypertension. The indications to perform a TIPSS procedure can be framed in an elective or emergency setting: refractory ascites to diuretic therapy and secondary prophylaxis of variceal hemorrhage are the most frequent reasons for executing a TIPSS in an election context, while acute uncontrolled variceal bleeding is the principal indication that a TIPSS needs to be performed in an emergency setting. In recent years, the role of the TIPSS has been redefined for several conditions, such as ectopic varices, portal vein thrombosis, Budd–Chiari syndrome, hepatic veno-occlusive disease, and many others. This review aims to perform a deep analysis of when and why a TIPSS procedure should be carried out in an emergency, pointing out the related most common technical difficulties and complications.

## 1. Introduction

The ultimate goal of a transjugular intrahepatic porto-systemic shunt (TIPSS) is to create a connection between the portal vein and the hepatic vein blood flow to reduce the pressure gradient in portal hypertension (PH) and, as a result, to lessen the clinical consequences of PH, such as ascites, oesophageal-gastric varices and hepatorenal syndrome, just to mention a few [1]. To find the first attempts at porto-systemic communication, we have to look back to the early 1960s, when Rösch and coworkers reported the incident of an incidental portography through a transjugular approach [2]. It was not until 1988 that the first human TIPSS procedure was performed by Richter et al. in Freiburg, Germany [3].

A TIPSS is usually performed in cirrhotic patients with a stable clinical condition; secondary prevention of esophageal variceal bleeding as well as refractory ascites to diuretic therapy represent the most common reasons to perform a TIPSS in an ordinary setting [4]. However, sudden clinical worsening of these patients is a frequent outcome, due to the underlying uncontrolled portal hypertension, leading to acute esophageal or gastric-esophageal variceal bleeding.

Apart from cirrhosis-related portal hypertension, a TIPSS can be a feasible treatment in many more situations, such as hepatorenal syndrome (both type 1—now defined as HRS-AKI, acute kidney injury—and 2), Budd–Chiari syndrome, hepatic veno-occlusive disease, hepatic hydrothorax, hepatopulmonary syndrome and portal vein thrombosis [5,6,7].

There are situations in which the interventional radiologist is required to decide whether a TIPSS is really necessary in emergency situations, and when it is advisable to postpone the procedure until a stable clinical situation has been reached. Unfortunately, literature on this topic is scarce; therefore, the purpose of this review paper is to discuss the role of the TIPSS in an emergency setting, with a focus on indications, patient selection criteria, technical aspects, and procedural difficulties during a TIPSS in an emergency setting.

## 2. Epidemiology and Outcomes of Emergency TIPSS

There are few studies in the literature that consider the incidence of a TIPSS in an emergency situation: among these, Bañares et al. reported a 15% rate of emergency TIPSS in patients with acute variceal bleeding not responding to pharmacological and/or endoscopic treatment (e.g., variceal sclerotherapy and ligature) [8]. Conversely, out of a total of 34 patients evaluated, the proportion of urgent TIPSSs documented in clinical practice by Goykhman et al. is higher, coming in at roughly 53% [9].

One reason for this paucity of data in relation to the TIPSS in an emergency setting can be found in the fact that TIPSS is a highly specialized procedure in the field of interventional radiology, with availability limited mostly to high-end centers [10]. For example, Thabut et al., in their study involving 964 participants, discovered that even if 35% of the patients met the requirements for a TIPSS in 72 h (p-TIPSS), only 6.8% of patients actually received it; moreover, as is shown in Dunne et al. ’s multicenter study, 55% of the 58 patients requiring a p-TIPSS had it completed within that time; the remaining patients did not undergo TIPSS at all [11,12].

Regarding TIPSS outcomes, Zhu et al. measured the mean transplant-free survival time after a TIPSS placement for acute variceal bleeding, with six-weeks, one-year and two-year rates of 87.7%, 81.8%, and 73.6%, respectively [13]. Goykhman et al. reported a 72% overall survival rate after an urgent TIPSS procedure, compared to a 100% overall survival rate after an “elective”, non-urgent TIPSS [9].

## 3. Pre-Procedural Work-Up

Proper execution of a TIPSS in an emergency setting requires a thorough clinical evaluation of the patient. The pre-procedural work-up must include: a clinical evaluation of the patient’s life expectancy, as well as the patient’s hemodynamic status, respiratory condition, and mental state; laboratory tests, particularly regarding the liver function, coagulation status, and kidney function; a recent and valid diagnostic imaging study (usually a triphasic contrast-enhanced CT scan or, at least, a Doppler ultrasound examination) to assess the patency of the portal vein and of the hepatic veins, and to exclude the presence of any liver lesion that might prevent the creation of the porto-systemic shunt; ruling out any significant medical conditions that would make the TIPSS contraindicated, such as severe liver failure, severe hepatic encephalopathy, severe pulmonary hypertension, severe right heart failure, numerous large hepatic cysts, or an unrelieved biliary obstruction [14]. Table 1 summarizes the most important contraindications to performing an emergency TIPSS. It must be clear that the TIPSS exerts an important effect in the passage of ammonium and other toxins directly into the systemic circulation, with a considerable risk of hepatic encephalopathy (90% in the first three months), especially if there is a prior history of hepatic encephalopathy. Moreover, a TIPSS increases the systemic venous blood return from the portal system towards the systemic circulation; therefore, patients with preclinical cardiac insufficiency as well as those who already have overt heart failure or severe tricuspid regurgitation may experience heart failure as a result of the post-TIPS spike in the preload [15].

The patient must have an underlying disease that can benefit from the TIPSS procedure; nevertheless, the operator needs to be certain that there is a good likelihood the patient will experience a clinical improvement after the procedure. Intraprocedural trans-jugular portal vein and hepatic vein pressure measurements are, therefore, mandatory to assess the porto-systemic gradient and the presence of portal hypertension, which can be relieved with the TIPSS procedure [16].

## 4. Indications for Emergency TIPSS

TIPSSs performed in an emergency setting are strictly time-dependent. The main indications are represented by the following pathologic events.

### 4.1. Esophageal Variceal Bleeding

Some 15–20% of patients with acute variceal bleeding (AVB) would exhibit failure to stop bleeding or early rebleeding within five days despite the use of “gold-standard” pharmaceutical and endoscopic therapy [17]. A TIPSS in the case of acute esophageal variceal bleeding can control the hemorrhage in 90% of patients, even though it has a one-month rebleeding rate of about 15% [18]. A TIPSS performed soon after the initial pharmacological and endoscopic treatment, within 72 h—or, better, within the first 24 h—of the start of the bleeding, a so-called “early TIPSS”, has been introduced as a treatment recommendation for PH since 2015 [19]. According to the definition of “early TIPSS”, the procedure is performed after a first-line endoscopic ligation approach failure [20]. Various studies have proven that patients with high-risk hemorrhage, in which HVPG (hepatic venous pressure gradient, a parameter used to assess portal vein pressure, with normal values between 3–5 mmHg) was higher than 20 mmHg, showed a significant improvement in bleeding control and survival rates after a TIPSS, particularly in the first year after the treatment, compared to patients treated with beta-blockers and an endoscopic band ligation. Of interest is the fact that hepatic encephalopathy (HE) presented with the same percentages across those studies, about 30% [21,22]. The literature recommends performing the so-called “early TIPSS” in high-risk patients (Child–Pugh class C with a score below 14) with no contraindications to a TIPSS (in particular, congestive heart failure, tricuspid regurgitation and serious pulmonary hypertension) [23,24]. In particular, among patients with advanced liver disease, Nicoară-Farcău et al. noticed that patients with a Child–Pugh score equal to 7 had a significantly better survival than those with a Child–Pugh score greater than 7 after a TIPSS (*p* < 0.0001) [25].

### 4.2. Gastric Variceal Bleeding

Approximately 20% of individuals with portal hypertension will develop gastric varices. Gastric varices have a lower probability of bleeding than esophageal varices but, when bleeding occurs, they require significantly more blood transfusions [26]. More specifically, fundal varices tend to have a higher fatality rate [27]. A TIPSS is successful in attaining hemostasis in 90–96% of instances where acute bleeding is unresponsive to vasoconstrictor and endoscopic treatment [28,29].

There are very few studies on the role of a TIPSS in acute gastric variceal bleeding. Procaccini et al. showed that individuals who underwent a TIPSS for an acute gastric variceal hemorrhage had rebleeding rates, hospitalization time, and one-year mortality rates comparable to those who underwent endoscopic treatment with cyanoacrylate glue [30]. The current recommendations suggest the use of a TIPSS for gastric varices with the highest risk of bleeding (e.g., varices in a cardiofundic position) [31]. In addition to a TIPSS, some authors recommend coil embolization of gastric varices, to maximize the curative effectiveness in the case of an acute hemorrage; however, it is still unclear whether a TIPSS and variceal coil embolization work better in combination when compared to a TIPSS alone [32,33,34]. Patients who continue to experience gastroesophageal bleeding even though they have had a functioning porto-systemic shunt may consider variceal coil embolization.

### 4.3. Less Frequent Indications for Emergency TIPSS

#### 4.3.1. Budd–Chiari Syndrome

Hepatic venous outflow obstruction due to thrombosis of the hepatic veins and/or the hepatic portion of the inferior vena cava characterizes Budd–Chiari syndrome (BCS), which can cause PH. Generally, anticoagulation is implemented as the first line of treatment for BCS, and decompressive therapies are used if there are any indications of PH. Angioplasty with or without stenting is the first interventional option, while a TIPSS is considered in the case of heavy hepatic vein obstruction [5,35]. In a retrospective study by Garcia-Pagán and colleagues, the one- and five-year liver transplant-free survival rates were 88% and 78%, respectively, in 124 patients with BCS who received a TIPSS [36]. Goykhman et al. reported a case of acute Budd–Chiari syndrome treated with a TIPSS which unfortunately had a poor outcome [9]. In a work by Blum et al., 12 out of 12 patients had an effective TIPSS placement, with an average decrease in the portal venous gradient of 75% and no significant procedure-related problems, even though the subsequent shunt malfunctioning, occurring in half of the patients, necessitated further treatment [37]. Because of these not fully concordant results and the relative lack of literature data due to the rarity of the BCS, further studies are mandatory for understanding the role of an emergency TIPSS in BCS.

#### 4.3.2. Hepatorenal Syndrome

In the absence of another cause, hepatorenal syndrome (HRS) is the development of renal impairment in cirrhotic patients, as renal vasoconstriction and renin-angiotensin-aldosterone pathway activation are the results of splanchnic vasodilation and circulatory dysfunction [38]. Among the two different forms of HRS, type 1 (now known as HRS-AKI, acute kidney injury) is characterized by the patient’s abrupt clinical decompensation. A TIPSS has been demonstrated to decrease plasma renin and aldosterone levels while increasing the glomerular filtration rate and salt excretion through the urine [39]. The application of a TIPSS in an HRS-AKI has not been examined in randomized controlled studies, although it is logically reasonable. Although patients who undergo a TIPSS have improved renal hemodynamics and renal function as well as fewer recurrences of HRS-AKIs in the follow-up, the placement of a TIPSS is contraindicated in classic HRS type 1 because it has been linked to cardiac and hepatic disease [40].

In a study by Brensing et al., 10 non-transplantable patients with HRS who had extensive liver failure that prevented a TIPSS placement were compared to 31 non-transplantable patients who received a TIPSS for HRS (14 HRS-AKI): the three-month survival rate in the TIPSS group was 81%, compared to 10% for patients who were not eligible for a TIPSS [41]. Although a TIPSS in this setting may avert persistent renal impairment and the need for further liver–kidney transplants, the clinical evidence on the use of a TIPSS for HRS is too limited and, therefore, a TIPSS is not usually proposed for this condition.

## 5. Timing of Emergency TIPSS

In the scenario of uncontrolled variceal bleeding, a TIPSS is characterized by a high level of effectiveness and applicability but, nonetheless, the ideal timing to carry out a TIPSS is still up for dispute, as the type of emergency, the timing of bleeding episodes, as well as the patient’s vital parameters affect the decision to intervene [42]. Where local resources allow, an early TIPSS should be considered within 72 h of a variceal bleeding. For example, failure of two consecutive endoscopic procedures is a simple and clear definition of uncontrolled variceal bleeding; however, this does not necessarily specify the requirements for a TIPSS implantation. A patient with a Child–Pugh class “A” often presents acute variceal bleeding without life-threatening characteristics (e.g., HVPG < 12 mmHg, with a low bleeding risk) and may be managed by balloon tamponade followed by further sessions of endoscopic ligation and, generally, does not require a TIPSS [43,44]. Conversely, patients who had a single, massive bleeding, with an unsuccessful endoscopic treatment which required balloon tamponade, may be better treated by a TIPSS rather than undergoing a second endoscopic therapy session [43].

## 6. Technical Difficulties of Emergency TIPSS

Classical technical aspects of a TIPSS placement procedure are beyond the aim of this paper, and have been thoroughly described by other authors [44,45]. Therefore, only procedure-related difficulties in emergency settings and tricks to avoid them will be described.

Ultrasound-guided percutaneous access to the internal jugular vein is the first step in a TIPSS insertion. The right internal jugular vein is usually preferred due to a better catheterization angle, although the left internal jugular vein can offer a better hepatic vein puncture angle and can also be used when the right one is occluded or already occupied by central venous catheters [46]. Although this is uncommon, care must be taken to avoid a carotid or subclavian artery puncture, as well as a lung apex puncture or puncture of other soft cervical tissues leading to pneumothorax and hematoma, particularly in patients with a low platelet count [47].

When advancing through the right atrium to gain access to the inferior vena cava and the hepatic veins, a cardiac arrhythmia may be brought on by the guidewire irritating the endocardium, utterly decompensating a critically unstable patient; however, a catheterism of the inferior vena cava is generally sufficient to interrupt the arrhythmia.

The choice of the best hepatic vein to catheterize is fundamental, and brings forth its own risk of complications: choosing a right inferior accessory hepatic vein (a venous anatomical variant present in nearly one-third of patients) may result in a transcapsular liver puncture during tract construction, with a risk of damaging the kidney, colon, gallbladder, or pancreas, and also a risk of intraperitoneal bleeding [48].

The right hepatic vein is the usual access for the creation of the TIPSS, but when the anatomy is unfavorable, TIPSS formation through the middle or left hepatic vein is a feasible alternative, even though it can be more time-consuming in an emergency setting [49]. Therefore, preprocedural treatment planning is of utmost importance.

In patients with Budd–Chiari syndrome, one or more hepatic veins are typically permanently blocked. In an emergency setting, it is possible to wedge the TIPSS needle into the right hepatic vein stump, exploiting the favorable angle given by a left internal jugular vein approach [48]. Otherwise, a direct puncture through the caval wall typically necessitates redesigning the cannula into a considerably larger angulation to allow a safe puncture.

Portal vein puncture should be performed only when the venous branches are clearly seen under ultrasound guidance, particularly in an emergency setting, to avoid serious complications [49].

Care must be taken when placing the TIPSS stent, as a too peripheral parenchymal tract can lead to the stent-graft’s angle being acute, without clinical improvement of the portosystemic pressure gradient [50].

## 7. Complication of Urgent TIPSS

Numerous issues may arise during or after the TIPSS implantation process, especially in emergency settings. Table 2 summarizes the most important relevant complications when performing a TIPSS, especially in emergency cases.

There are two main types of inconveniences: intraprocedural and post-procedural, the latter being subdivided into an early phase and delayed phase.

### 7.1. Intraprocedural Complications

Lesions to the hepatic artery (about 1% of cases), bile duct (about 5% of cases), or liver capsule leading to hemoperitoneum, parenchymal hemorrhage, pseudoaneurysm development, acute hepatic artery thrombosis/spasm, artero-portal fistula, hemobilia due to artero-biliary or porto-biliary fistulae, and/or lesions to adjacent organs (e.g., kidney, gallbladder, bowel) may occur during TIPSS needle passes [51].

During TIPSS needle passes, 33% of patients may experience liver capsule transgression, whereas 1–2% of patients develop intraperitoneal bleeding [52]. Care must be taken to assess for hemoperitoneum, particularly if arterial enhancement is seen during the puncture procedure, requiring an urgent arterial embolization [53]. Patients should be treated for a potentially life-threatening hemorrhagic complication if they show signs and symptoms of intraprocedural bleeding, such as hemodynamic instability, hematemesis, increased abdominal discomfort, or growing abdominal distension. If cone-beam CT is available, it can be quickly used to determine whether bleeding is present and where it is located, assisting a prompt direct embolization therapy. If the bleeding is of arterial origin, an angiography must be performed as soon as possible, and embolizing agents or covered stents should be used to control this complication [54]. On the other hand, if the bleeding is of venous origin, considering that damage to the main, left, and right portal veins cannot be effectively addressed with embolization, a successful TIPSS insertion represents the most effective therapy.

The gallbladder is the organ that is most frequently damaged when the liver capsule is penetrated, leading to hemobilia, cholangitis, and/or an intrabiliary clot. The right kidney, duodenum, and colon’s hepatic flexure are additional organs that can suffer from off-target punctures. Such organ punctures are often well tolerated, with only a few occurrences of clinically important sequelae being described (e.g., parenchymal hematoma, abscess formation, bowel perforation) [53,54].

The emergency setting requires particular attention to avoid the unintentional deployment of the stent when inserting it into the vascular introducer sheath. There are two moments when an unintended stent opening may occur: the first situation is the incorrect withdrawal of the clear plastic sleeve that restricts the endoprosthesis, because the sleeve is readily confused with the protective covering that would typically be removed from an angioplasty balloon catheter. The second possibility, probably more frequent, happens when the introducer sheath’s hemostatic valve is being passed through by the transparent sleeve that is holding the bare end of the stent-graft in place. A partial misdeployment of the endoprosthesis into the hub of the sheath can happen if the plastic sleeve is not fully inserted into the sheath until the black marker line aligns with the sheath valve; this prevents the stent-graft from being advanced past the hub into the sheath [55].

### 7.2. Early Postprocedural Complications

Liver rupture and intra-abdominal bleeding are two of the most common early postprocedural issues which may occur after a TIPSS implantation. Liu et al. reported the case of a patient who developed arterial bleeding with associated hepatic laceration eight days after the TIPSS placement, while other patients experienced intrahepatic hematoma two weeks after the TIPSS. At the time of the bleeding, all patients received either warfarin or low-molecular-weight heparin [56,57,58,59,60]. In these cases, a contrast-enhanced CT examination should be undertaken to determine the existence and location of the bleeding, and anticoagulant and antiplatelet drugs should be discontinued. Hepatic angiography is the definitive diagnostic and therapeutic resource in the case of bleeding of arterial origin.

One of the most common complications which can arise shortly after a TIPSS begins to properly function is represented by hepatic encephalopathy (HE). HE can occur as early as one day after the TIPSS insertion or as late as 210 days after. Until a few years ago, most cases of hepatic encephalopathy were treated using branched-chain amino acids and a low-protein diet [61]. At present, the combined use of rifaximin and lactulose represents the treatment of choice for hepatic encephalopathy, greatly limiting low-protein diet-related muscle mass wasting [62]. A total of 1–3% of TIPSS patients may develop severe, debilitating encephalopathy, up to a condition of cerebral edema (if ammonia levels exceed 70 μg/dL), resulting in the risk of diffuse ischemic cerebral damage if the TIPSS is not revisioned [63]. The diameter of the stent-graft can be reduced and the most commonly used way to reduce the stent-graft diameter is represented by the placement of an hourglass-shaped stent-graft in the site of the previous stenting, or by using similar devices [64,65,66].

To address severe HE, a TIPSS can also be occluded, using an angioplasty balloon for at least 12 h or with a vascular plug, to obtain thrombosis; however, after the TIPSS was closed significant hemodynamic alterations were reported, which eventually led to patient death, as TIPSS closure (both desired and undesired) usually determines the rapid recovery of the pre-TIPSS conditions due to portal hypertension, such as ascites and variceal hemorrhage [67,68].

Early acute stent occlusion can be seen in up to 5% of patients [69]. After the TIPSS placement, bile duct-to-TIPSS fistulas can be quite troublesome, leading to acute TIPSS thrombosis due to the thrombogenic effect of the bile; however, the use of coated stents limits this issue [70].

Even when the covered stent placement is correctly performed, there is still the risk of stent thrombosis and/or stenosis [71]. While performing a TIPSS, the association of variceal embolization or sclerotherapy offers another set of possible complications. The risk of stent thrombosis increases with non-selective embolization of varices by coils, vascular plugs, glue, or sclerosing agent [72]. Coils or n-butyl-cyanoacrylate glue can eradicate smaller varices, but much bigger varices may need considerably higher amounts of embolizing agent (up to 50 mL), with the risk of pulmonary embolism if it enters the pulmonary circulation. A recent study by Chen et al. on 134 patients determined that variceal embolization added to the TIPSS creation did not significantly decrease the rate of rebleeding in cirrhotic patients after a TIPSS [73].

Much more usual is stent thrombosis due to improper stent placement, usually because the hepatic end of the stent-graft can be located in the parenchyma or can lean against the vessel wall; in this way, the flow via the TIPSS is impeded, leading to TIPSS malfunctioning and thrombosis [74]. Even though portal venography performed immediately after the TIPSS insertion may demonstrate acceptable stent configuration, the removal of the guidewire or catheter may alter its configuration, leading to a compromised flow that looks very different from the final portal venogram.

When thrombosis develops, the treatment of acute TIPSS obstruction frequently involves a mix of angioplasty, thrombolysis (which is carried out by local catheter-directed infusion with lytic agents such as the tissue-type plasminogen activator), and thrombectomy [75]. When all these approaches are in vain, it can be useful to catheterize the TIPSS again, overcoming the thrombosis, to place a new stent-graft inside. The final alternative is to create a new TIPSS.

Following the TIPSS implantation, some patients could develop acute hepatic failure, as a TIPSS placement will significantly lower the portal pressure, as far as reversing the portal venous flow, theoretically causing some degree of ischemia. In addition, the TIPSS may compress or obstruct the hepatic artery and/or portal vein branches, causing ischemia or infarction. If not correctly placed, the covered section of the TIPSS has the potential to occlude one or more hepatic veins, leading to abrupt liver failure and hepatic ischemia similar to Budd–Chiari’s [76,77]. It is mandatory to preemptively evaluate this procedural risk in individuals with significant coagulopathy and altered liver function. It is recommended that the most adequate portosystemic gradient be obtained, because a significant decline may put patients at risk of developing acute liver failure. Generally, it is not advised to have a post-TIPSS portosystemic gradient of less than 5 mmHg [77]. If there is no indication of vascular occlusion or thrombosis, the changed portal venous flow dynamics may be the most likely cause of acute liver failure, necessitating an immediate attempt to occlude the TIPSS. However, eventually, an immediate liver transplantation could be the only cure for this terrible condition.

### 7.3. Delayed Postprocedural Complications

An important late complication is represented by stent migration. The risk of stent migration is usually about 1%, and is mostly associated with the use of bare-metal stents [78]. Inferior vena cava (IVC) thrombosis, atrial perforation, aorto-atrial fistula, and cardiac arrhythmia can all result from the stent migrating into the IVC, in the right heart or in the pulmonary artery [79]. In addition, when performing a liver transplant, clamping the IVC may become more challenging due to the stent’s misplacement. A similar issue arises when the stent migrates caudally into the portal system, necessitating repair of the primary portal vein and, if necessary, of the superior mesenteric or splenic vein [53]. Although stent repositioning can be tried using balloons or snares, surgical removal of the migrated stent is the only effective therapeutic option. One patient was reported to be asymptomatic at six years with the stent-graft migrated into the heart, suggesting that non extraction of an intracardiac TIPSS may be possible [78].

Between weeks and months after the TIPSS procedure, patients may manifest an infectious state with persistent bacteremia without a defined cause: in such a case, the possibility of a stent-graft infection (also known as endotipsitis) should be considered. This is a rare complication (about 1% incidence according to Kochar et al.) which may be characterized by a severe clinical course if not quickly identified [80]. Cholangitis and biliary venous fistulas are frequently linked to endotipsitis. Contrast-enhanced CT or MR does not provide a definitive diagnosis, although they can help rule out other various infection etiologies. In the case of a stent-graft infection, as the stent is usually unremovable, the only available treatments are represented by liver transplantation or lifelong antibiotics.

Variceal late rebleeding after a TIPSS is a reality, with a poor outcome [81]. Luo et al. reported a two-year rebleeding rate of 22.2% after a TIPSS [82]. Rebleeding following a TIPSS is typically caused by a TIPSS blockage [83]. In these circumstances, it is mandatory to confirm the TIPSS’s patency: a Doppler ultrasound examination and/or a contrast-enhanced CT scan with direct assessment of the stent represents the gold standard. If deemed necessary, a transjugular TIPSS evaluation with a potential thrombectomy can be useful. Variceal embolization should also be considered, if feasible [84]. Some studies have shown that within two years of the TIPSS implantation, 70 to 90% of patients require reintervention to restore or maintain the patency of the shunt [85].

Table 3 summarizes the most important intraprocedural, early, and delayed complications of the TIPSS placement.

## 8. Discussion

First-line therapy for bleeding varices, represented by nonselective beta-adrenergic blockers alone or associated with endoscopic variceal ligation is effective in 80% of cases; in the remaining percentage, intraesophageal balloon tamponade and the implantation of esophageal self-expandable metal stents (SEMS) can be considered as bridging therapies to a TIPSS and liver transplantation [18]. The most popular type of balloon tamponade, the Sengstaken–Blakemore tube, has a 90% hemostasis rate; complication rates vary between 20–60% according to the time of placement; therefore, its use should be restricted to bleeding occurring after less than 24 h. Esophageal rupture or ulceration, aspiration pneumonia, or asphyxia, are a few of the side effects of the balloon tamponade. Compared to the balloon tamponade, and the Sengstaken–Blakemore tube in particular, SEMS have higher success rates, better bleeding control, less need of blood transfusion, less significant adverse events, and lower TIPSS conversion rates; however, the risk of esophageal ulceration, stent migration, rebleeding at 48 h, as well as rebleeding after stent removal is high with SEMS. Therefore, it is advised that the stent be removed within seven days of its placement [86,87].

Concerning the success rate of a TIPSS performed in the emergency state, multiple systematic reviews and meta-analyses have evaluated the safeness and the feasibility of the procedure and the percentage of survival over time.

A meta-analysis by Guo et al. showed an efficient rebleeding prevention and good survival rates in cirrhotic patients: a rebleeding rate of about 13%, with hepatic encephalopathy in 32% of cases, 80% of overall survival [88]. In a meta-analysis, Weichselbaum et al. compared 23 studies for a total of 1430 patients, finding that about two-thirds of patients survived after the first six weeks of a TIPSS placement [89].

Furthermore, research by Lv et al. was primarily concerned with reducing the risk of liver transplantation or mortality in cirrhotic patients who experienced acute variceal bleeding. They discovered a 13% absolute risk decrease at one year (95% CI) [90]. According to Urata et al., patients with a 50% reduction in the initial HVPG had a one-year rebleeding risk of 11%, whereas those with a lower degree of HVPG reduction had a 31% chance of doing so [91]. Additionally, in the context of refractory ascites, Rossle et al. observed that patients waiting for a liver transplantation are more likely to survive on the waiting list when undergoing a TIPSS (69% and 58% at one and two years, respectively) than when treated with repeated paracentesis (52% and 32% at one and two years, respectively) [92].

An interesting finding is that, according to the work conducted by Perarnau et al., there is no significant difference between older bare metal stents and more recent covered ones in terms of overall survival and HE [71]. Regarding HE, a TIPSS leads to the development of HE with similar rates (35–33%) between different studies; however, HE is a small complication when compared to the extremely high risk of a patient’s death if a TIPSS is not performed as a salvage therapy [13].

## 9. Conclusions

Emergency TIPSS is a life-saving procedure for patients with hepatopathy leading to uncontrolled variceal bleeding. Bleeding prevention with an early TIPSS should be routinely performed when possible, because it avoids the critical escalation of this dangerous and life-threatening pathological condition. Even though multiple studies seem to assess the superiority of a TIPSS compared with the best actual first-line medical and endoscopic therapies, more data are needed to understand the real risks and benefits of this procedure, particularly in the emergency setting [43]. Indeed, the outcomes of placing a TIPSS in urgent settings other than acute bleeding, such as in a hepatopulmonary syndrome, need to be evaluated. Regarding the use of a TIPSS as a bridge to liver transplantation, several studies demonstrated the effectiveness of this procedure in different settings.

Furthermore, to conclude, it would be useful to understand the relationship between certain biomarkers (if any) and the degree of stent dysfunction as well as the patient’s survival rate [93].

## Figures and Tables

**Table 1:** Contraindications to perform an emergency TIPSS.

Absolute	Relative
Congestive heart failure [15]	Portal vein thrombosis [14]
Severe tricuspidal regurgitation [15]	Severe coagulopathy [15]
Severe pulmonary hypertension [15]	Hepatic encephalopathy [14]
Severe systemic sepsis [14]	Hepatocellular carcinoma [14]
Unrelieved biliary obstruction [14]	Budd–Chiari syndrome [14]

**Table 2:** Indications for emergency TIPSS.

Acute Variceal Bleeding	Budd–Chiari Syndrome	Hepato-Renal Syndrome
First-line therapy: drugs plus endoscopy (unsuccessful in 15–20% of AVB) [17]	First-line therapy: anticoagulants +/− hepatic vein angioplasty or stenting	TIPSS decreases renin and aldosterone plasma levels, increasing GFR and salt excretion [39]
TIPSS recommended for PH-related AVB (AVB control in 90% of cases) [18]	TIPSS considered in case of significant hepatic vein obstruction (HVPG reduction in 75% of cases) [35,37]	Increase in 3-month OS [41]
Significant symptoms improvement in patients with HVPG > 20 mmHg with similar HE incidence [21,22]	Lack of literature data	Lack of literature data

AVB, active variceal bleeding; TIPSS, transjugular intrahepatic porto-systemic shunt; PH, portal hypertension; HVPG, hepatic venous pressure gradient; HE, hepatic encephalopathy; GFR, glomerular filtration rate; OS, overall survival.

**Table 3:** Emergency TIPSS: intraprocedural, early, and delayed complications.

Intraprocedural	Early Postprocedural	Delayed Postprocedural
Hemoperitoneum [51]	Liver rupture [56]	Stent migration [78]
Artery thrombosis [53]	Hepatic encephalopathy [61]	Endotipsitis [80]
Porto-biliary fistula [51]	Early stent occlusion [69]	Variceal rebleeding [81]
Lesion adjacent organs [51]	Liver failure [76]	Late stent blockage [83]

## Data Availability

Not applicable.
